# Does Round-Ligament-Based Non-Mesh Pectopexy Provide Durable and Effective Apical Support After Total Laparoscopic Hysterectomy?

**DOI:** 10.3390/jcm15134912

**Published:** 2026-06-24

**Authors:** Mehmet Yaman, Kevser Arkan

**Affiliations:** 1 Department Obstetrics and Gynecology, Diyarbakir Gazi Yasargil Research and Training Hospital, 21090 Diyarbakir, Turkey; 2 Division of Gynecologic Oncology, Department Obstetrics and Gynecology, Diyarbakir Gazi Yasargil Research and Training Hospital, 21090 Diyarbakir, Turkey; kevser.toprak1989@gmail.com

**Keywords:** non-mesh pectopexy, round ligament suspension, apical prolapse, total laparoscopic hysterectomy, vaginal cuff support, vaginal length, sexual function, minimally invasive gynecologic surgery

## Abstract

**Objective:** To assess the anatomical and clinical outcomes of a novel, mesh-free cerclage pectopexy technique that uses the round ligament for apical support following total laparoscopic hysterectomy in women with stage II uterine prolapse. **Methods:** This retrospective observational study included 120 women with stage II uterine prolapse who underwent total laparoscopic hysterectomy followed by laparoscopic non-mesh pectopexy between October 2023 and August 2024. In this procedure, the distal portion of each round ligament was fixed to the pectineal ligament using Ethibond sutures. Multiple plicating stitches were then placed to reinforce the ligament’s tensile strength, creating a biological suspension bridge between the pectineal ligament and the vaginal cuff. All patients were examined preoperatively and at 1, 3, 6, and 12 months postoperatively using the POP-Q system. Anatomical success was defined as an apical stage ≤ I at 12 months. **Results:** At the twelve-month follow-up, anatomical success was achieved in 95 percent of patients, with six cases of apical recurrence. POP-Q measurements showed significant improvement from baseline, and total vaginal length was preserved. Functional outcomes, including postoperative pain and dyspareunia, were favorable. Early complications were uncommon, and no intraoperative or mesh-related complications occurred. **Conclusion:** Round-ligament-based non-mesh cerclage pectopexy provides reliable apical support with minimal surgical morbidity following total laparoscopic hysterectomy. This technique appears to provide effective apical support with low surgical morbidity while avoiding synthetic mesh. Preservation of vaginal length and favorable short-term clinical outcomes were observed; however, longer-term comparative studies are required. Future prospective studies combining this procedure with other minimally invasive suspension techniques, such as McCall culdoplasty or uterosacral plication, may broaden its applicability to more advanced prolapse cases.

## 1. Introduction

Restoration of apical support remains one of the most critical determinants of long-term success in pelvic reconstructive surgery. In anterior colporrhaphy, the most frequently performed reconstructive procedure, the absence of adequate apical suspension has been shown to increase the likelihood of prolapse recurrence and the need for reoperation [1]. Apical suspension can be achieved through transabdominal or transvaginal approaches using either native tissue or synthetic mesh materials. Previous biomechanical studies have demonstrated that the pectineal (Cooper’s) ligament is structurally stronger and more resistant than the sacrospinous ligament or the arcus tendineus fascia pelvis [2].

Several studies have shown that sacrocolpopexy is the most suitable approach for restoring the physiological vaginal axis in terms of size, depth, and inclination [3,4]. However, positioning the mesh between the sacrum and the vagina can lead to narrowing of the pelvis—particularly in obese patients where prolapse is more common—and may cause technical difficulties. In such cases, fatty deposits around the sigmoid colon can further restrict the operative space for mesh placement, increasing the likelihood of postoperative pain, defecatory dysfunction, and bowel symptoms resembling diverticulosis [5]. During the procedure, the sigmoid colon, hypogastric nerves, and the right ureter are also at risk of injury. Compared with the abdominal route, the laparoscopic approach offers a clear advantage by improving postoperative recovery without increasing the overall complication rate [6].

For these reasons, Banerjee and Noé first introduced laparoscopic pectopexy (LP) in 2011, using bilateral pectineal ligaments as anchoring points for synthetic mesh fixation [7]. This modification allowed the ureters and bowel to remain safely outside the surgical field, avoiding presacral dissection and reducing the risk of injury. Subsequent studies have confirmed that laparoscopic pectopexy is an effective treatment for apical pelvic organ prolapse [8,9]. Nonetheless, the use of synthetic mesh has been associated with complications such as infection, erosion, chronic pain, and mesh-related injury to adjacent organs, limiting its broader use. Moreover, in patients without a uterus, apical support in pelvic organ prolapse can often be achieved without the need for a dense synthetic material that elicits a strong inflammatory response.

To address these limitations, Zhang and colleagues (2022) proposed a novel non-mesh cerclage pectopexy technique that is performed bilaterally after supracervical hysterectomy using sequential sutures placed through the cervix, round ligament, and pectineal ligament [10]. They reported a 100% objective and 93.8% subjective success rate at 6-month follow-up, defined respectively as the absence of prolapse beyond the hymen and a Patient Global Impression (PGI) score ≤ 2. Unlike the original technique described by Zhang et al., which was performed after supracervical hysterectomy, the present modification incorporates the vaginal cuff into the suspension pathway following total laparoscopic hysterectomy. To our knowledge, this is the first clinical series evaluating this adaptation in women undergoing total hysterectomy.

## 2. Methods

### 2.1. Study Design and Ethical Approval

This single-center retrospective observational study was conducted at the Department of Obstetrics and Gynecology, Diyarbakır Gazi Yaşargil Training and Research Hospital. The study protocol was reviewed and approved by the Institutional Ethics Committee of Diyarbakır Gazi Yaşargil Training and Research Hospital (Approval No: 231; Approval Date:11 October 2024). Written informed consent was obtained from all participants prior to inclusion. The present study represents a retrospective analysis of routinely collected clinical data from patients who underwent standard surgical care. No intervention was performed for research purposes, and patient management was not influenced by study participation. The ethics committee reviewed and approved the use of anonymized clinical data in accordance with institutional policies and the Declaration of Helsinki.

The primary objective of the study was to evaluate the anatomical and selected clinical outcomes of a round-ligament-based non-mesh cerclage pectopexy performed simultaneously with total laparoscopic hysterectomy (TLH) in women with stage II uterine prolapse.

### 2.2. Patient Selection and Eligibility Criteria

This retrospective observational study included 120 women with stage II uterine prolapse who underwent total laparoscopic hysterectomy followed by laparoscopic pectopexy at our institution between October 2023 and August 2024. All patients were evaluated preoperatively and at 1, 3, 6, and 12 months postoperatively [11]. Among women undergoing total laparoscopic hysterectomy for benign gynecologic indications such as leiomyoma, adenomyosis, abnormal uterine bleeding, or similar non-malignant conditions, those with concomitant POP-Q stage II uterine prolapse identified during preoperative evaluation were included in the study. Non-mesh pectopexy was performed to provide apical support following hysterectomy. Eligible participants were required to have stage II uterine prolapse based on the POP-Q classification [12], no prior pelvic malignancy. Only patients with complete clinical records and a minimum of 12 months of available follow-up were included in the final analysis. Patients were excluded if they had a history of pelvic reconstructive surgery or prior mesh use, if they presented with POP-Q stage III or IV prolapse, or if any factor was identified that could introduce procedural heterogeneity. Patients with stage III–IV prolapse were excluded because these patients are routinely managed with alternative surgical approaches at our institution, most commonly vaginal hysterectomy combined with site-specific reconstructive procedures. Including these patients would have introduced substantial heterogeneity in both surgical technique and baseline disease severity, thereby limiting the ability to evaluate the performance of the non-mesh pectopexy technique in a uniform cohort.

### 2.3. Surgical Technique

All surgeries were performed laparoscopically by the same senior surgical team. The procedures were performed by the same senior surgical team consisting of experienced gynecologic oncologists and advanced laparoscopic surgeons. Ethibond No. 1 sutures were routinely used; however, in two cases, a nonabsorbable suture with lower tensile resistance was used due to temporary material limitations, without altering the surgical steps. Operative time was measured from first incision to skin closure, excluding anesthesia induction.

After completion of a standard TLH, the vaginal cuff was closed using a V-Loc barbed suture, and the pectopexy procedure was initiated [13]. During hysterectomy, the round ligaments were meticulously preserved, maintaining approximately two-thirds of their length attached to the pelvic sidewall [7].

**Figure 1:**
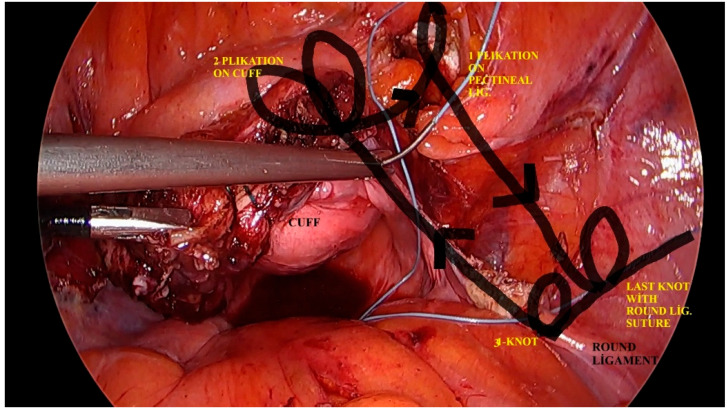
Intraoperative illustration of the cerclage pectopexy suspension pathway. A laparoscopic intraoperative image demonstrating the key anatomical landmarks involved in the procedure. A schematic overlay is used to delineate the trajectory of the Ethibond suture on the right side, showing the sequential passage through the distal round ligament, the lateral edge of the vaginal cuff, and the pectineal ligament. The colored line highlights the completed suspension vector, illustrating the final configuration of the anchoring system.

The pectopexy technique consisted of the following standardized sequence:

1. Initial fixation at the distal round ligament:
  The distal segment of each round ligament was identified. An Ethibond No. 1 suture was passed transversely through the ligament and secured with one surgical knot followed by one simple knot. Routine plication of the ligament was not performed; however, short-segment plication was selectively applied only when the ligament was excessively long or the tissue appeared highly elastic [2].
2. Suspension through the vaginal cuff:
  The needle-bearing long limb of the same Ethibond suture was passed twice through the lateral edge of the sutured vaginal cuff in a manner resembling plication, while keeping the ureteral course under direct visualization. No knot was tied at this stage, and gentle traction was applied to remove slack.
3. Anchoring at the pectineal ligament:
  The same needle was directed to the ipsilateral pectineal ligament, previously exposed by limited blunt dissection. A single firm pass was made through the ligament to achieve stable anchorage. The suture was tightened to eliminate any redundancy, creating a preliminary suspension line between the cuff and pectineal ligament.
4. The long limb of the suture was held under controlled traction with a laparoscopic grasper to prevent relaxation. It was then tied to the short distal limb originating from the round ligament using one surgical knot followed by a simple knot, consistent with previously described knot sequences [14], completing the apical suspension construct, as illustrated in Figure 1.

**Figure 2:**
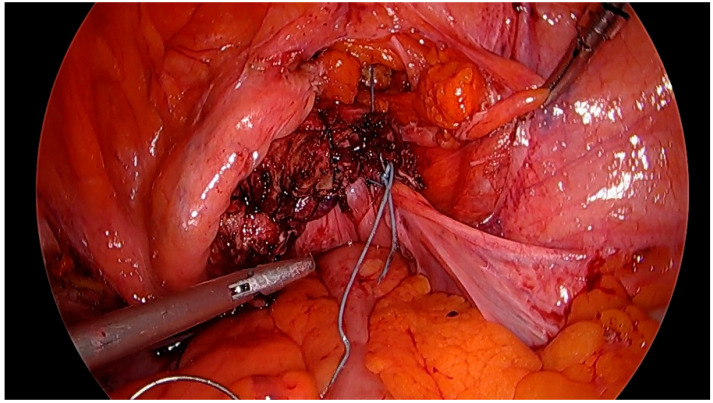
Final anatomical configuration after completion of right-sided pectopexy. Laparoscopic view demonstrating the postoperative alignment of key pelvic structures following suspension on the right side. The round ligament is shown secured to the pectineal ligament with the completed Ethibond suture construct, with the suture line also incorporating the lateral edge of the vaginal cuff.

The same sequence was repeated on the contralateral side to achieve symmetrical apical support. The final unilateral anatomical configuration is shown in the intraoperative image presented in Figure 2.

5. At the end of the procedure, the peritoneum covering the operative site was closed with 3-0 Vicryl sutures to restore peritoneal integrity and reduce adhesion formation. The sutures were intentionally left slightly taut to compensate for physiological tissue relaxation after pneumoperitoneum release and potential gradual elongation of the round ligament over time [15].

**Figure 3:**
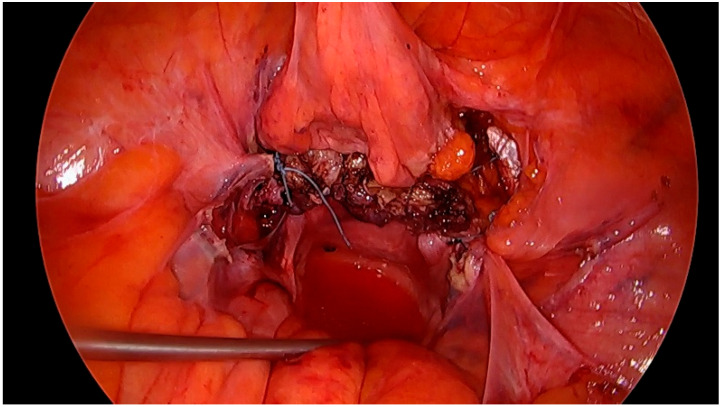
Bilateral cerclage pectopexy before peritonization: final apical position of the vaginal cuff. Laparoscopic view obtained after completion of right and left pectopexy and before peritoneal closure.

This non-mesh technique created a biological suspension bridge between the round ligament, the vaginal cuff, and the pectineal ligament, eliminating the need for synthetic mesh and thereby avoiding mesh-related complications such as chronic pelvic pain and erosion [16]. The final bilateral apical configuration after completion of the suspension is demonstrated in Figure 3. A step-by-step operative demonstration of the technique is provided in Supplementary Video S1.

Perioperative data collection included a comprehensive set of demographic and surgical variables such as age, parity, body mass index (BMI), history of previous pelvic surgery, and the type of surgical approach used for the concomitant procedure. The operative approach was classified as laparoscopic (LS), laparotomy (LT), or vNOTES. Additional variables included operative time, intraoperative blood loss, length of postoperative hospital stay, and intraoperative or postoperative complications. Anatomical evaluation was performed using the POP-Q system by comparing the points Aa, Ba, C, D, Ap, Bp, and total vaginal length (TVL) in the preoperative and postoperative assessments [12]. Anatomical success was defined as the vaginal apex being positioned at or above the hymenal level (Stage ≤ I) at the twelve-month follow up [12]. Functional outcomes were assessed by measuring postoperative pain at 6 and 24 h using the visual analog scale (VAS) [17], recording the duration of analgesic requirement, and documenting early complications such as urinary retention, bleeding and infection. Late complications, including dyspareunia, chronic pelvic pain and voiding or defecatory dysfunction, were also recorded, while “mesh erosion” was noted as zero in all cases due to the non-mesh nature of the technique. Recurrence of apical prolapse was additionally evaluated as part of the functional outcome assessment.

### 2.4. Surgical Equipment and Materials

Procedures were performed using a standard four-port laparoscopic configuration with a 10 mm umbilical trocar for the camera and three 5 mm accessory ports. Tissue dissection and hemostasis were performed using energy devices (LigaSure™, Medtronic, Minneapolis, MN, USA) and bipolar instruments.

Suturing materials included Ethibond™ No. 1 (Ethicon, Somerville, NJ, USA) for suspension and 3-0 Vicryl™ for peritoneal closure. The vaginal cuff was closed using a V-Loc™ barbed suture (Covidien, Mansfield, MA, USA). Detailed information on the suture materials, energy devices, and laparoscopic equipment used during the procedure is provided in Supplementary Table S1.

### 2.5. Statistical Analysis

Continuous variables were summarized as mean ± standard deviation (SD) or median (interquartile range [IQR]), depending on distribution, and categorical variables as frequency and percentage. Pre- and postoperative POP-Q parameters were compared using repeated-measures linear mixed-effects models; when assumptions were unmet, the Friedman test or Wilcoxon signed-rank test was applied.

Anatomical success and complication rates were expressed with 95% confidence intervals (CIs) using the Wilson method. Independent predictors of anatomical failure were examined using multivariate logistic regression, including variables such as age, BMI, parity, prior pelvic surgery, operative duration, blood loss, surgical approach, and suture material. Results were reported as odds ratios (ORs) with 95% CIs. All tests were two-sided with *p* < 0.05 considered significant. Analyses were performed using IBM SPSS Statistics v26.0 (IBM Corp., Armonk, NY, USA).

## 3. Results

A total of 120 women with stage II uterine prolapse who underwent total laparoscopic hysterectomy followed by laparoscopic non-mesh pectopexy were included. The mean age was 57.7 ± 8.9 years, the mean body mass index was 29.6 ± 3.8 kg/m^2^, and the mean parity was 3.4 ± 1.2. The conversion rate to laparotomy was 0.0% (0/120). The post-operative blood transfusion rate was 6.6% (8/120). No intraoperative transfusions were required due to acute hemorrhage. All eight transfusions were administered post-operatively to manage symptomatic anemia triggered by standard surgical blood loss in patients with pre-existing low baseline hemoglobin due to chronic abnormal uterine bleeding. The mean operative time was 100.8 ± 14.3 min, the mean estimated blood loss was 159.7 ± 42.6 mL, and the mean postoperative hospital stay was 1.4 ± 0.6 days (Table 1).

At the twelve-month follow-up, anatomical success—defined as apical POP-Q stage I or better—was achieved in 114 of 120 patients (95.0 percent). Six patients (5.0 percent) experienced apical recurrence. Two of these recurrences occurred in cases where an alternative nonabsorbable suture with lower tensile strength had been used due to a temporary limitation in material availability. This suggests that the reduced durability of the substitute suture may have contributed to partial loss of apical support, rather than indicating a weakness of the surgical technique itself. The remaining four recurrences occurred in patients with satisfactory intraoperative findings and no identifiable technical deviation. None of the patients required reoperation during the follow-up period.

**Figure 4:**
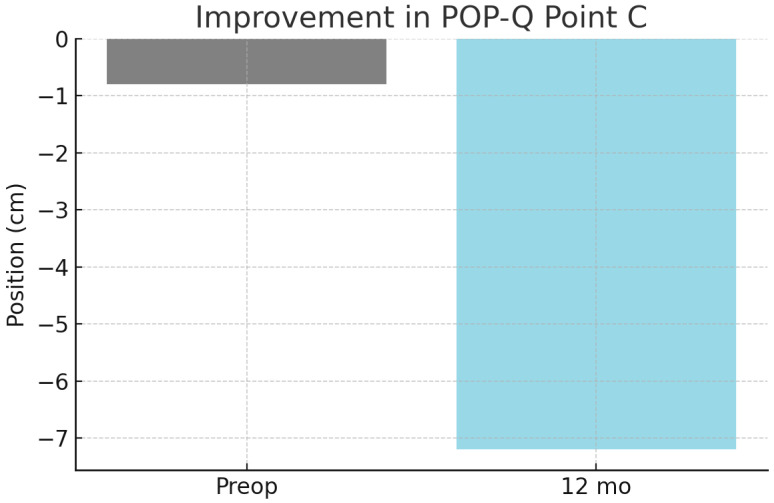
Change in POP-Q point C position before and after surgery. Comparison of the apical position (point C) measured by the Pelvic Organ Prolapse Quantification (POP-Q) system before surgery and at 12-month follow-up. Bars represent the mean values (±standard deviation). A significant upward shift in point C from −0.8 ± 1.0 cm preoperatively to −7.2 ± 0.9 cm at 12 months (*p* < 0.001, Wilcoxon signed-rank test) indicates durable restoration of apical support after the round-ligament-based non-mesh cerclage pectopexy procedure.

All POP-Q parameters showed significant improvement compared with baseline (*p* < 0.001) (Table 2). Median point C improved from 0.8 cm above the hymen preoperatively to 7.2 cm above the hymen at the twelve-month follow up, as illustrated in Figure 4. The mean total vaginal length was preserved, measuring 6.1 ± 0.7 cm preoperatively and 6.3 ± 0.8 cm postoperatively (*p* = 0.42).

Postoperative pain scores were low, with mean VAS values of 1.1 ± 0.8 at 6 h and 0.9 ± 0.6 at 24 h. The mean duration of analgesic use was 1.6 ± 0.8 days. Early minor complications occurred in 12 patients (10.0%), most commonly transient urinary retention (*n* = 4, 3.3%). No intraoperative bladder, ureteral, or bowel injuries were observed.

During follow up, six patients (5.0%) developed small cuff epithelial erosions at the suture line, all resolving with topical estrogen therapy. Dyspareunia was reported by 10 patients (8.3%), and chronic pelvic pain by 2 (1.6%); both rates were lower than those reported in mesh based pectopexy series. No new onset voiding or defecatory dysfunction occurred (Table 3). However, urinary symptoms, including occult stress urinary incontinence, were not systematically recorded. This limitation precludes a comprehensive assessment of urinary outcomes.

Outcomes did not differ significantly between surgical approaches (LS, LT, or vNOTES) or according to prior pelvic surgery history (*p* > 0.05 for all). In multivariate logistic regression, age, BMI, parity, previous pelvic surgery, operative time, blood loss, surgical approach, and suture material were not independent predictors of anatomical failure.

No statistically significant association was observed between these variables and postoperative anatomical success (*p* > 0.05 for all, logistic regression).

## 4. Discussion

Restoration of apical support remains a key objective in pelvic organ prolapse surgery, as it is one of the most effective strategies for reducing recurrence. With the growing adoption of laparoscopic approaches, surgeons continue to seek techniques that improve anatomical success while minimizing patient morbidity. Traditional sacrocolpopexy offers durable long-term outcomes. However, the presacral placement of mesh introduces important risks, including bowel injury, defecatory dysfunction, chronic pelvic pain, and nerve damage [18]. Because of these concerns, there has been renewed interest in mesh-free apical suspension techniques in recent years.

The laparoscopic pectopexy technique described by Banerjee and Noé in 2011 provides a safer alternative by securing apical support through the pectineal ligament, thereby eliminating the need for presacral dissection [7,14,19]. However, the use of synthetic mesh in this procedure has been associated with complications such as infection, erosion, and chronic pelvic pain [20]. These limitations have prompted interest in achieving apical suspension with a biologically compatible, mesh-free approach that could maintain durable support without foreign material.

In 2022, Zhang and colleagues reported a non-mesh cerclage pectopexy technique performed bilaterally after supracervical hysterectomy, in which sequential sutures were placed through the cervix, round ligament, and pectineal ligament [10]. Their study demonstrated 100% anatomical and 93.8% subjective success rates at 6-month follow-up. This approach leveraged the inherent strength of the pectineal ligament while introducing a biological suspension model that obviated the need for mesh. Our study builds upon this innovative concept by demonstrating the feasibility of a round-ligament-based non-mesh pectopexy performed after total laparoscopic hysterectomy. In these patients, where the removal of the cervix further compromises apical support, the interposition of the round ligament serves as a biological bridge that restores structural stability. Unlike series utilizing supracervical fixation, our approach demonstrates cuff security without cervical tissue; however, since pectopexy shifts the vault more ventrally than sacropexy, it should be viewed as providing an anatomically favorable support vector rather than perfectly preserving the physiological axis.

The technique used in our study offers both physiological and mechanical advantages. After closure of the vaginal cuff, the distal portion of the remaining round ligament was anchored with Ethibond sutures, and short-segment reinforcement plication was selectively applied in cases where the ligament was excessively long or demonstrated marked elasticity to enhance its tensile support over time. The sutures were intentionally left slightly taut to compensate for the relaxation expected once intraabdominal pressure decreases at the end of laparoscopy. This biomechanical adjustment represents a key innovation distinguishing the current method from conventional pectopexy. By utilizing the round ligament as a biological intermediary between the pectineal ligament, which serves as a strong anchoring point, and the vaginal cuff, which represents the structurally weaker apical tissue, a natural suspension bridge was created that eliminates the need for synthetic mesh.

At the twelve-month follow-up, our study demonstrated a 95.0 percent anatomical success rate, which is comparable to outcomes reported in mesh-based pectopexy series [14]. Complication rates were low, and operative morbidity remained minimal. The absence of mesh-related complications further supports the safety of this non-mesh approach. Two early recurrences were associated with the temporary use of a suture material with lower tensile strength, suggesting that these events reflected material variation rather than a failure of the technique. This observation underscores the importance of maintaining consistency in suture selection to ensure uniform tension and durable apical support. The low incidence of dyspareunia and chronic pelvic pain may be attributed to the elimination of direct mesh contact with vaginal tissues, thereby reducing irritation. In addition, the physiological elasticity of the round ligament likely contributes to faster postoperative recovery and improved functional outcomes.

The main strength of our study lies in the high level of technical standardization and the fact that all procedures were performed by the same surgical team. The inclusion of patients with stage II prolapse allowed an objective evaluation of the technique within a homogeneous cohort. Nevertheless, the retrospective design of the study limits its generalizability. Because only patients with complete clinical records and 12-month follow-up data were included in the analysis, the possibility of selection bias cannot be excluded. An additional limitation is the lack of validated patient-reported outcome measures, including quality-of-life and patient satisfaction assessments. Given the retrospective design, standardized questionnaires were not routinely collected. Therefore, although anatomical success was achieved in most patients, the relationship between anatomical correction and patient-reported benefit could not be fully evaluated. Future prospective studies should incorporate validated patient-reported outcome instruments. In addition, the small number of anatomical failure events limited the statistical power of the multivariable analysis. Therefore, the absence of significant predictors of recurrence should be interpreted with caution. Finally, the follow-up period was limited to 12 months. Although the short-term outcomes were favorable, the long-term durability of the procedure remains uncertain and requires confirmation in studies with extended follow-up.

Restricting the study population to patients with stage II prolapse was a deliberate decision aimed at assessing the safety and efficacy of the procedure under uniform conditions. While this choice enhanced internal validity, it also limits the direct applicability of the findings to advanced prolapse stages. Future prospective studies evaluating non-mesh pectopexy in stage III-IV prolapse, either through vaginal or combined approaches, would provide valuable clinical insight and help define the broader role of this technique in apical prolapse repair.

Another noteworthy aspect of this technique is that it imposes minimal surgical burden on the surrounding tissues, both in terms of the materials used and the operative steps required. The combination of the inherent strength of the pectineal ligament and the elastic suspension provided by the round ligament ensures strong apical support without the use of mesh. The absence of synthetic material eliminates foreign body reactions and tissue irritation, thereby facilitating smoother postoperative healing. For these reasons, round-ligament-based non-mesh pectopexy can be regarded as a low morbidity procedure that shortens operative time and enhances postoperative comfort.

In addition, the minimally invasive nature of this approach allows the potential for combination with other low-trauma suspension techniques to enhance apical support in more advanced prolapse cases. The literature includes several reports describing the combination of pectopexy with procedures such as McCall culdoplasty, uterosacral ligament plication, or sacrouterine fixation to reinforce apical suspension [21,22,23]. McCall culdoplasty strengthens the posterior compartment of the pelvic vault, whereas uterosacral plication maintains the vaginal axis in a more physiological alignment, promoting long-term stability. These combined strategies, particularly in patients with stage III prolapse or a widened genital hiatus, may provide more durable apical support when used together with the round-ligament-based pectopexy.

Therefore, the non-mesh pectopexy technique described in our study offers a modifiable surgical platform that can be adapted for advanced prolapse cases by combining it with other minimally invasive procedures, such as McCall culdoplasty or uterosacral plication. The technique’s low tissue trauma and high biological compatibility make it particularly suitable for individualized surgical planning. In this respect, it may represent a step toward a more personalized and physiologically oriented approach to pelvic organ prolapse repair in the future.

In conclusion, round-ligament-based non-mesh pectopexy performed after total laparoscopic hysterectomy appears to be a safe and effective technique for achieving durable apical support, with favorable anatomical and short-term clinical outcomes. By combining the firm anchoring capacity of the pectineal ligament with the inherent elasticity of the round ligament, this method restores apical support without the use of mesh and provides a mesh-free alternative for apical support following hysterectomy, although longer-term comparative studies are needed to confirm durability and functional outcomes. Accordingly, the present study contributes to the growing body of evidence supporting biologically compatible, low-complication, and potentially long-lasting surgical solutions for apical prolapse repair.

## Figures and Tables

**Table 1:** Demographic and perioperative characteristics of the study population (*n* = 120).

Variable	Mean ± SD
Age (years)	57.7 ± 8.9
Body mass index (kg/m^2^)	29.6 ± 3.8
Operative time (min)	100.8 ± 14.3
Estimated blood loss (mL)	159.7 ± 42.6
Postoperative hospital stay (days)	1.4 ± 0.6
Parity (*n*)	3.4 ± 1.2

Values are presented as mean ± standard deviation or *n* (%), unless otherwise specified.

**Table 2:** Changes in Pelvic Organ Prolapse Quantification (POP-Q) parameters before and after surgery.

POP-Q Parameter	Preoperative Mean ± SD	Postoperative (12 Mo) Mean ± SD	*p* Value
Aa	1.8 ± 0.7	−2.7 ± 0.5	<0.001
Ba	1.9 ± 0.6	−2.9 ± 0.6	<0.001
C	−0.8 ± 1.0	−7.2 ± 0.9	<0.001
Ap	1.5 ± 0.7	−2.4 ± 0.5	<0.001
Bp	1.4 ± 0.8	−2.5 ± 0.7	<0.001
TVL	6.1 ± 0.7	6.3 ± 0.8	0.42

Values are presented as mean ± standard deviation or *n* (%), unless otherwise specified. POP-Q = Pelvic Organ Prolapse Quantification system; TVL = total vaginal length. Differences between preoperative and postoperative values were analyzed using the Wilcoxon signed-rank test. A *p* value < 0.05 was considered statistically significant.

**Table 3:** Complications and functional outcomes.

Outcome	*n* (%)
Anatomical success (Stage ≤ I at 12 mo)	114 (95.0%)
Early minor complications	12 (10.0%)
Urinary retention	4 (3.3%)
Cuff epithelial erosion	6 (5.0%)
Dyspareunia	10 (8.3%)
Chronic pelvic pain	2 (1.6%)
New onset voiding dysfunction	0 (0.0%)
Defecatory dysfunction	0 (0.0%)
Post-operative blood transfusion	8 (6.6%) *

Values are presented as mean ± standard deviation or *n* (%), unless otherwise specified. All complications were classified according to the Clavien-Dindo system. Anatomical success was defined as apical POP-Q Stage ≤ I at 12 months. No major intraoperative complications occurred. *: Transfusions were administered post-operatively to treat symptomatic anemia arising from the physiological drop in hemoglobin in patients with low pre-operative baselines, not due to active intraoperative surgical hemorrhage.

## Data Availability

The datasets generated and analyzed during the present study are available from the corresponding author on reasonable request.

## Supplementary Materials

The following supporting information can be downloaded at: https://www.mdpi.com/article/10.3390/jcm15134912/s1, Video S1: Operative demonstration of round-ligament-based non-mesh pectopexy, including step by step suture placement and final apical suspension configuration; Table S1: Surgical materials and equipment used in the round-ligament-based non-mesh pectopexy procedure.

## Abbreviations

BMI	Body mass index
IQR	Interquartile range
PGI	Patient Global Impression scale
PL	Pectineal ligament
POP-Q	Pelvic Organ Prolapse Quantification
RL	Round ligament
SD	Standard deviation
TLH	Total laparoscopic hysterectomy
TVL	Total vaginal length
VAS	Visual analog scale
VC	Vaginal cuff
vNOTES	Vaginal natural orifice transluminal endoscopic surgery
