# Supplementary material for: Does Round-Ligament-Based Non-Mesh Pectopexy Provide Durable and Effective Apical Support After Total Laparoscopic Hysterectomy?
Source: J Clin Med. 2026 Jun 24;15(13):4912. doi: 10.3390/jcm15134912 (PMC13360618; doi:10.3390/jcm15134912)
Supplement: Supplementary file 1 [file jcm-15-04912-s001.zip › jcm-4302359-supplementary.pdf]

Table S1. Surgical materials and equipment used in the round-ligament-based non-mesh pectopexy procedure

Category	Material/Equipment	Manufacturer (City, Country)
Suspension suture	**Ethibond™ Excel No. 1 polyester suture**	**Ethicon Inc., Somerville, NJ, USA**
Peritoneal closure suture	**Vicryl™ 3-0 polyglactin 910 suture**	**Ethicon Inc., Somerville, NJ, USA**
Vaginal cuff closure	**V-Loc™ 180 barbed absorbable suture**	**Medtronic/Covidien, Mansfield, MA, USA**
Vessel sealing system	**LigaSure™ Maryland jaw device**	**Medtronic, Minneapolis, MN, USA**
Bipolar coagulation system	**Bipolar electrosurgical forceps**	**Karl Storz SE & Co. KG, Tuttlingen, Germany**
Laparoscope	**10-mm 30° rigid laparoscope**	**Karl Storz SE & Co. KG, Tuttlingen, Germany**
Camera system	**High-definition laparoscopic camera system**	**Karl Storz SE & Co. KG, Tuttlingen, Germany***
Umbilical trocar	**10-mm trocar**	**Karl Storz SE & Co. KG, Tuttlingen, Germany**
Accessory trocars	**Three 5-mm trocars**	**Karl Storz SE & Co. KG, Tuttlingen, Germany***
Needle holder	**Laparoscopic needle holder**	**Karl Storz SE & Co. KG, Tuttlingen, Germany**
Grasper	**Atraumatic laparoscopic grasper**	**Karl Storz SE & Co. KG, Tuttlingen, Germany**

NJ, New Jersey; MA, Massachusetts; USA, United States of America.
